# Nanoplastic Pollution of Human Bronchoalveolar Lavage Probed Based on Tip-Enhanced Raman Scattering

**DOI:** 10.3390/s26154927

**Published:** 2026-08-04

**Authors:** Alberto Chaves, Grace Binder, Patrick Foti, Sugriva Forsyth, Eduardo Celis, Jaskaran S. Sethi, Tien Dao, Dawson Dodd, Kathleen M. Egan, Dmitri V. Voronine

**Affiliations:** 1 Department of Biomedical Engineering, University of South Florida, Tampa, FL 33620, USA; 2 Department of Physics, University of South Florida, Tampa, FL 33620, USA; 3 Department of Chemistry, University of South Florida, Tampa, FL 336204, USA; 4 Department of Cancer Epidemiology, Moffitt Cancer Center, Tampa, FL 33612, USA; 5 Department of Thoracic Oncology, Moffitt Cancer Center, Tampa, FL 33612, USA; 6 Department of Computer Science, University of South Florida, Tampa, FL 33620, USA

**Keywords:** nanoplastics, microplastics, bronchoalveolar lavage, Raman spectroscopy, TERS

## Abstract

Raman spectroscopy is a commonly used label-free technique for the chemical analysis of microplastics (MPs), which are defined as plastic particles larger than 1 μm but smaller than 5 mm size; given its small signal strength and diffraction-limited spatial resolution, Raman spectroscopy has limited applicability in the study of nanoplastics (NPs), which are defined as particles smaller than 1 μm. Tip-enhanced Raman scattering (TERS) is a promising technique that can overcome these limitations by using a single plasmonic tip of an atomic force microscope (AFM) to enhance the Raman signal from a small sample volume. Here we used TERS for the analysis of NPs in human bronchoalveolar lavage (BAL) fluid. We developed a sub-sampling procedure for nanoscale TERS imaging on an SiO_2_/Si substrate and performed control experiments. We investigated the experimental coffee-ring effect on the spatial distribution of NPs on the substrate. We compared the results of TERS imaging with the conventional confocal Raman microscopy and observed the presence of similar types of plastic, pigments, and mineral particles, revealing the origin of NPs from the corresponding MPs. The total nanoparticle density observed in BAL using TERS was ~50 billion particles per liter, most of which were NPs. Our TERS approach may be extended to other types of human fluids and tissue samples to provide insights into the health effects of NPs.

## 1. Introduction

Microplastic (MP) and nanoplastic (NP) pollution is present in the environment as manufactured MPs/NPs or from the continuous weathering of plastic litter, which results in progressively smaller nonbiodegradable plastic fragments [1] that may be ingested [2,3] and inhaled [4]. In the lung, MPs have been identified in lung tissue [5,6,7] and in sputum [8] and bronchoalveolar lavage (BAL) fluid [9,10,11]. Recent simulations predict deposition of both MPs and NPs in the upper and lower airways [12]. However, the health impacts of such internal MP/NP exposures are unknown [13,14,15,16]. Given the potential of MPs/NPs driving carcinogenesis through local inflammatory and immune responses or via direct exposure to chemical contaminants, chemosensitizers, and pathogens, there is an urgent need to fully characterize MP/NP exposures and determine whether the MP/NP burden is a risk factor for cancer in body sites such as the lung.

BAL is a minimally invasive procedure routinely performed during flexible bronchoscopy. BAL involves the installation of saline into the lower airway, the recovery of the instilled volume, and a diagnostic evaluation of the recovered BAL fluid. Prior studies have documented the utility of BAL as a minimally invasive approach to isolate MPs in the airways of living persons [9,10,11]. However, there have not yet been any thorough studies of NPs in BAL, mainly due to the lack of proper experimental techniques.

The various experimental techniques for studying MPs/NPs are summarized in Table 1. The main methods for the detection, identification, and quantification of MPs/NPs are based on scanning electron microscopy (SEM), pyrolysis-GC/MS, Fourier transform infrared (FTIR) spectroscopy, and Raman spectroscopy [17,18]. The existing methods for NP detection offer morphological or chemical information, but not both. For example, SEM can directly visualize NPs with high spatial resolution down to ~1 nm [19,20]. However, SEM offers limited information on the chemical composition of the particles, is not quantitative, and requires extensive sample preparation via Au coating to avoid space-charging effects on non-conductive substrates, which is destructive and prohibits further study using other methods. On the other hand, mass spectrometry-based techniques such as pyrolysis-GC/MS are destructive and provide no morphological information [21,22]. In GC/MS, the samples can be filtered only once prior to analysis, providing an upper bound to the NP size, and the method does not provide quantitative morphological information on the number of NPs or their size distribution. Table 1 shows the microscopy-based current methods and compares their main performance characteristics versus the TERS method.

FTIR [23,24] and Raman [18,25] microscopy are common spectroscopic methods for MP analysis. Both methods consist of two phases: first, physical characterization of the particles via optical microscopy, and second, chemical characterization of the particles via vibrational spectroscopy. For each identified particle, the laser is focused, and a spectrum is obtained on one or more spots on the particle surface. Both FTIR and Raman spectroscopy methods have different technical limitations: FTIR is subject to interference from water and may not be suitable for the study of MPs in aqueous and some biological samples [23]. On the other hand, Raman spectroscopy is subject to interference by fluorescence from different sources, including fluorescence from the substrate and pigments, and requires the preprocessing of samples to remove fluorescent background before Raman analysis [26]. Membrane filters or substrates may also introduce background in the FTIR of microplastics [27]. The corresponding spatial resolutions of ~10 µm (FTIR) and ~1 µm (Raman) are limited by diffraction.

TERS is a nanoscale extension of confocal Raman microscopy that enhances weak Raman signals and improves spatial resolution [28,29,30]. It is based on the resonant excitation of the collective electronic oscillations in a plasmonic gold-coated AFM tip acting as a nano-antenna and focusing light at the tip apex with intensity exceeding that of the incident light by several orders of magnitude. This enables the detection and imaging of small sample volumes down to a single-molecule level. This technology improves conventional Raman detection by several orders of magnitude. This also makes TERS imaging faster compared to conventional Raman imaging, which could take many hours for the same task that TERS can achieve in a few minutes. TERS is a combination of Raman spectroscopy and AFM topographic imaging. Therefore, this technology enables both the detection and counting of NPs (Table 1). TERS is non-destructive and minimally invasive in contrast to other techniques such as pyrolysis-GC/MS, which are destructive and cannot count NPs. TERS has another significant advantage compared to conventional Raman spectroscopy in that it can suppress the fluorescence background that is commonly observed in white, dark, and colored samples through a plasmonic effect of the proximity of the fluorescent dye to a gold surface [31]. This solves one of the major drawbacks of conventional Raman spectroscopy for NP analysis. Previous proof-of-principle TERS experiments on NPs were performed on spiked samples of isolated NPs [32,33]. However, no TERS studies have yet been performed on native (non-spiked) human or environmental samples.

In this work, we demonstrated the first application of TERS to human bronchoalveolar lavage for the detection and chemical analysis of NPs. We compared the chemical compositions of the MPs and NPs found using the conventional Raman and TERS methods, respectively. This work provides a validation study that suggests the applicability of TERS for NP detection within BAL samples for the future study of cancer and other thoracic diseases. We discussed the possible origins of BAL pollution from medical and laboratory environments.

## 2. Materials and Methods

BAL samples were obtained from patients referred for BAL at the Moffitt Cancer Center (MCC), Tampa, Florida, as part of an ongoing study investigating MPs as a novel risk factor for primary neoplasms of the lung. Persons with suspected new primary lung cancer, another primary neoplasms, or lung infection were eligible for inclusion. Bronchoscopy/BAL was carried out according to the standard practice at MCC. For purposes of the study, BAL was collected into 30 mL glass containers with metal lids that were washed with detergent, rinsed with ultrapure water, and dried under a fume hood with a lid in place prior to BAL fluid collection. Once collected, BAL samples were stored as-is without aliquoting or processing in a −80 °C freezer at MCC and transferred in batches to the Raman lab for MP/NP analysis. Measures were taken to minimize MP contamination from the OR and laboratory. In the lab, environmental MP pollution was minimized by rinsing all glassware several times with ultrapure water (Millipore, Bedford, MA, USA), and all BAL sample handling was performed under a ventilation hood.

Deposition of BAL droplets on Si/SiO_2_ substrates was performed via direct drop casting of the 10 µL droplets of the BAL fluid samples onto Si substrates with a 300 nm SiO_2_ coating that were precleaned with ethanol and air-dried under a fume hood. The BAL fluid samples were digested using a 10% KOH aqueous solution for 48 h and were filtered using a 1 µm pore size glass fiber filter (Whatman, Maidstone, UK). The remaining supernatant was then used as a source of the 10 µL droplets. We used this standard digestion method since previous literature reported multiple tests of the chemical stability and recovery of MPs/NPs in BAL and other samples [2,34]. We also performed our own recovery test on 30 µm PS spheres and did not observe any sample degradation.

Optical, Raman, AFM, and TERS measurements were performed using our setup that was previously described [35,36]. Briefly, AFM, Raman and TERS imaging were performed using a confocal microscope (LabRam, Horiba Scientific, Montpellier, France) coupled to a scanning probe microscope (OmegaScopeR, Horiba Scientific, Montpellier, France) with 638 nm laser excitation using an objective lens (NA = 0.9). AFM measurements were performed in tapping mode with a 20 nm average tip-sample distance. We checked the samples for thermal damage after every experiment through repeated AFM imaging. No significant changes in AFM sample morphology were observed. The radius of the laser focal spot was ~500 nm. Using a backscattering configuration with a 638 nm edge filter, the scattered signals were collected and detected using a spectrometer with a 600 g/mm grating coupled to a CCD camera. Raman maps were obtained using an acquisition time of 5 s and 0.5 mW laser power. Background subtraction in Raman spectral processing was done using 9th order polynomial fitting. All experiments were performed under ambient conditions at room temperature. The particle polymer and additive compositions were identified using IDFinder software (Horiba Scientific), which has a library of ~10,000 Raman spectra. A typical match index of >70% was used. Manual verification was additionally performed for ambiguous matches by visually comparing the spectral overlaps of the data with the database spectra.

## 3. Results

**Figure 1:**
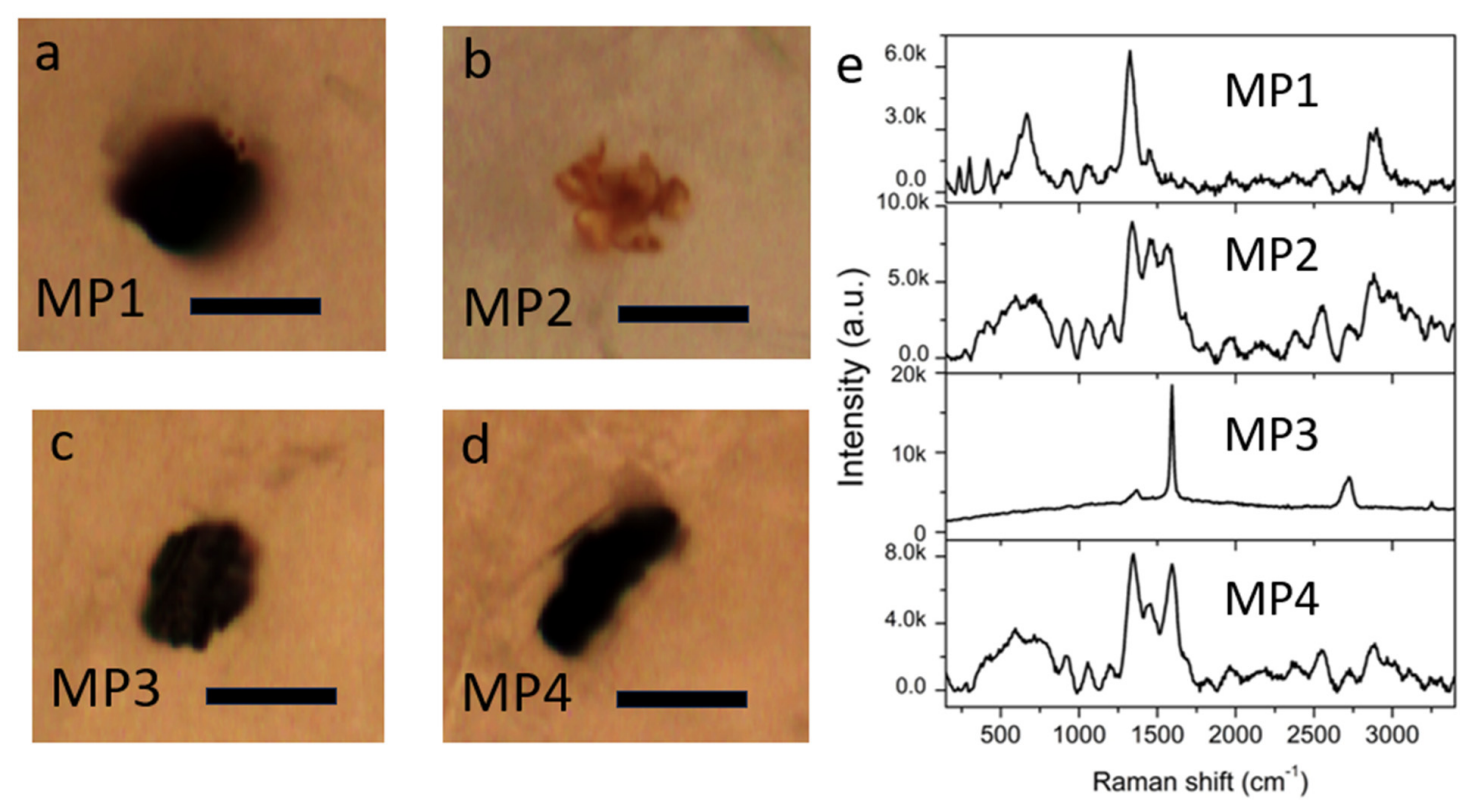
Microparticle identification via optical imaging and Raman spectroscopy. (**a**) MP1: hematite/PVC. (**b**) MP2: PE/PP. (**c**) MP3: graphite. (**d**) MP4: Irisol blue. Scale bar is 20 µm. (**e**) Raman spectra of MP1–MP4.

To demonstrate a proof-of-principle application of TERS to BAL fluid analysis, we randomly selected a BAL sample collected as part of the protocol described in the Methods Section. We digested 10 mL of BAL fluid in KOH and filtered it with a 1 µm-pore-size glass fiber filter. We then placed the dried filter in a Raman microscope and analyzed the whole filter area for MPs. We identified eight particles and obtained their optical images and Raman spectra. The particles were classified into MPs (polyethylene/polypropylene (PE/PP) and polyvinyl chloride (PVC)), minerals (hematite, graphite) and pigments (Irisol blue). The optical images and Raman spectra of four typical particles from human BAL fluid that included MPs, pigment, and mineral particles are shown in Figure 1. The typical particle size was ~10–20 µm. Dark and colored particles had a significant fluorescence background that was removed via polynomial fitting, as described in the Methods Section, leaving occasional residual wavy traces, as can be seen in the spectra in Figure 1e. We identified pure (for example, PE/PP) and composite aggregates of MPs and minerals, such as MP1 in Figure 1a (hematite/PVC).

**Figure 2:**
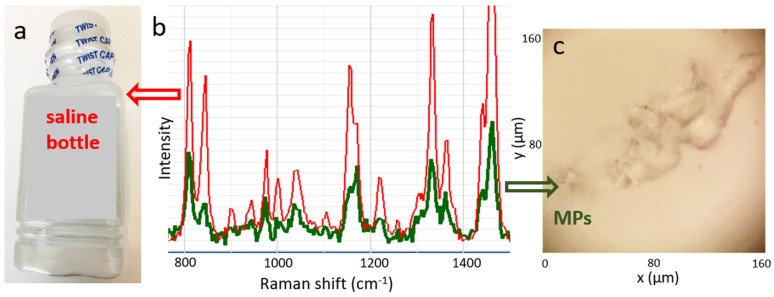
Control experiments on the plastic saline bottle used for the BAL procedure. (**a**) Photograph of the saline bottle. (**b**) Raman spectra of the saline bottle (red) and MPs found by filtering the saline solution through a 1 µm-pore glass fiber filter (green). (**c**) Optical image of the MPs from the filtered saline solution.

Control experiments with purified water were performed under identical conditions, and microparticles were also detected that were identified as PE/PP, hematite, and graphite. Therefore, the possible environmental contamination of the BAL fluid samples cannot be excluded. However, it was beyond the goals of the present work to investigate the MP environmental contamination. The goal of the present controls was to demonstrate the validity of the conventional Raman microscopy and to compare it with TERS. Another type of control was performed on the pure saline solution, without administering it to the patient. The resulting Raman spectra of the plastic saline bottle and MPs found in saline are in good agreement (Figure 2). The Raman spectra show a significant match that confirms the presence of MP background pollution in the control saline solution and identifies a specific contaminant from this source.

**Figure 3:**
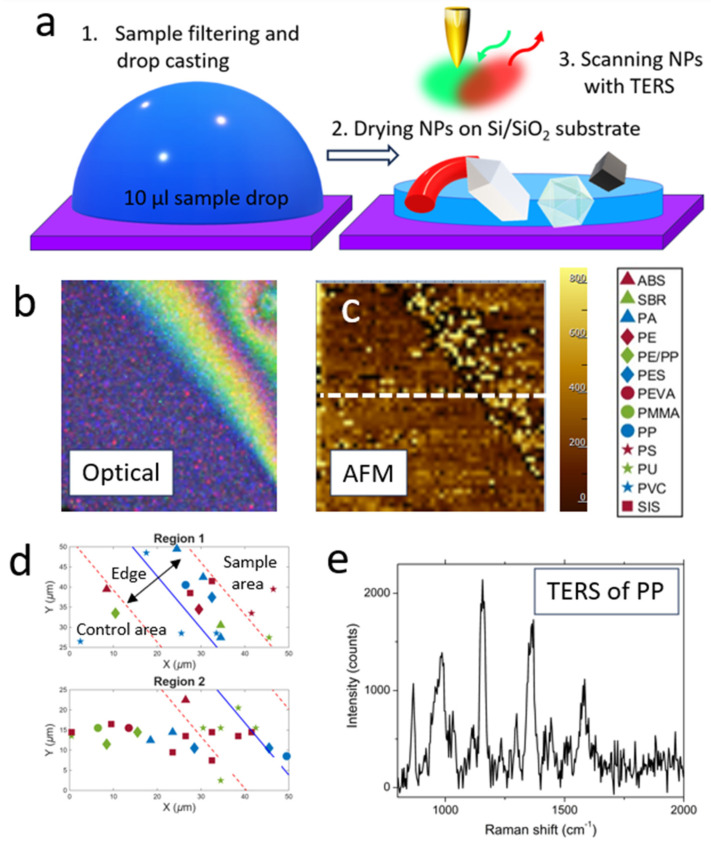
TERS of NPs. (**a**) Schematic of a three-step procedure for imaging of NPs using TERS. A droplet of filtered sample is drop-cast and dried on an SiO_2_/Si substrate, and a selected square area on the edge of the drop (**b**,**c**) is scanned via AFM/TERS. (**b**) Optical image and (**c**) AFM image of the scanned areas. The white dashed line in (**c**) separates the AFM area into two regions, shown in (**d**). The resulting TERS spectra (**e**) revealed the presence of more than 60 NPs shown by various colors and symbols in (**d**) with the corresponding abbreviated plastic assignments (inset labels): acrylonitrile butadiene styrene (ABS), styrene-butadiene rubber (SBR), polyamide (PA), polyethylene (PE), polypropylene (PP), polyester (PES), polyethylene vinyl acetate (PEVA), polymethyl methacrylate (PMMA), polystyrene (PS), polyurethane (PU), polyvinyl chloride (PVC), styrene-isoprene (SIS).

TERS experiments were conducted on the filtered BAL samples from which MPs larger than 1 µm were excluded, as described above. Figure 3a shows the schematic three-step sample-preparation process for the TERS measurements. NPs were characterized using TERS on 10 µL droplets air dried on SiO_2_/Si substrates, which were chosen as standard substrates that are background-free and minimize the fluorescence/Raman background common for other substrates, such as glass or quartz. The lateral droplet size after drying was approximately 1 mm^2^, which is too large for scanning the whole area with nanoscale resolution. For example, a scan of a 1 mm × 1 mm area with a 1 µm step size will generate 1 million Raman spectra, which will take 2 weeks to measure with a 1 s acquisition time. Therefore, a subsampling procedure was used, where two smaller areas of 50 µm × 25 µm were randomly selected on the edge of the sample drop as shown in the optical and AFM images in Figure 3b and Figure 3c, respectively. Scanning these areas with a step size of 1 µm generated 2500 TERS spectra in 42 min and identified more than 60 nanoparticles, almost half of which were NPs. The locations of NPs are shown in Figure 3d with the labels in the inset of Figure 3. We analyzed two different regions shown as region 1 and region 2 in Figure 3d across the different edge positions of the droplet. We estimated the total NP burden of 18 and 23 particles from regions 1 and 2, respectively. This corresponds to 3 and 13 NPs in the control areas of regions 1 and 2, respectively. These results indicate significant spatial heterogeneity, which can be addressed in large-scale future work. An example of the polypropylene (PP) TERS spectrum is shown in Figure 3e. TERS spectra showed a large signal-to-noise ratio (SNR) that had a high match index in the identification spectral library, as described in the Methods Section.

AFM and TERS measurements were performed simultaneously with the same step size of 1 µm. Therefore, the quality of the AFM imaging was low due to the large step size of 1 µm during the scan resulting in only 50 data points in the 50 µm range. It was not possible to detect NPs using AFM due to the additional morphological signals from the contamination of the BAL sample by other biological and inorganic nanoparticulate matter that passed through the filter but had a weaker Raman spectrum and did not comprise NPs. On the other hand, TERS has a different imaging contrast that is based on the spectroscopic, rather than morphological, signals, and it was able to clearly distinguish the NP signals from the weaker non-NP background. The AFM height scan in Figure 3c showed a clear edge of the dried BAL drop. The edge line determined from the AFM image was used as a guide to separate the scanned area into the sample (S) and control (C) areas with and without the BAL fluid, respectively. We chose an uncertainty of 20 µm and defined it as the width of the edge (E) area shown by the double-sided arrow in Figure 3d. We compared the E and S areas to investigate the “coffee ring” effect of the result of a drying drop, which often leads to a high concentration of NPs at the drop edge [37,38].

**Figure 4:**
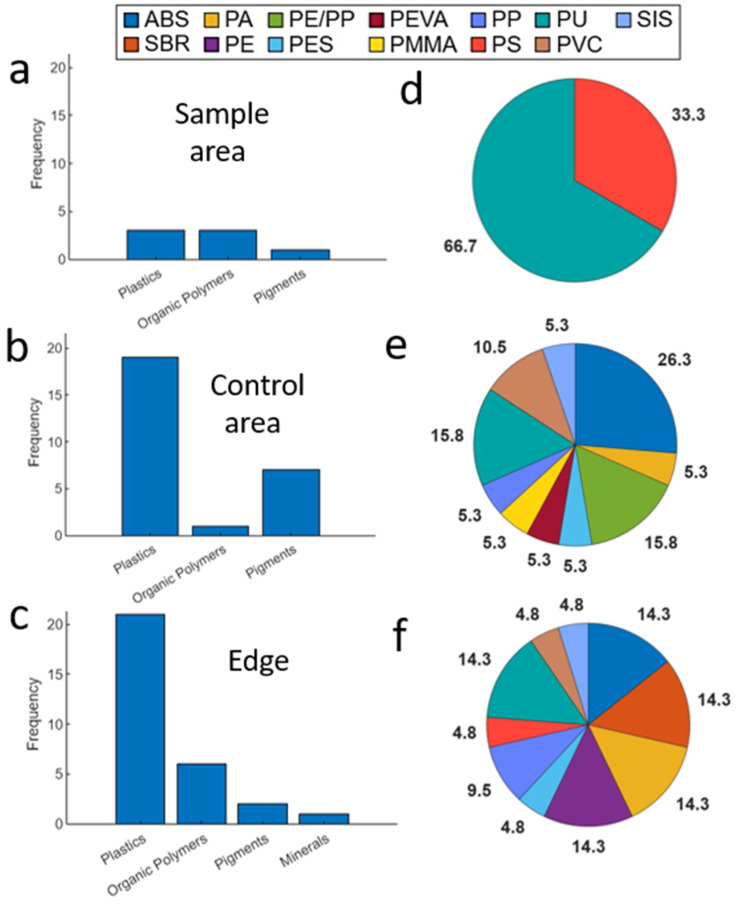
Summary of the TERS results: occurrence frequencies (**a**–**c**) and relative NP concentrations (**d**–**f**) from the (**a**,**d**) BAL sample, (**b**,**e**) SiO_2_/Si control, and (**c**,**f**) BAL drop edge areas.

For the TERS experiments, it is crucial to perform the control measurements under identical conditions during the same scan with the same tip. Therefore, we were able to compare the S, E, and C areas from the same scan. The results are summarized in Figure 4 and Table 2. The detected nanoparticles were classified into four groups, namely plastics, organic polymers, pigments, and minerals. Their occurrence frequencies and relative NP concentrations in the three areas are shown in Figure 4a–c and Figure 4d–f, respectively.

To eliminate a possible interference effect on the control measurement from the BAL sample, we performed another control experiment on a clean SiO_2_/Si substrate without BAL (Table 3). The comparison of the results in Table 2 and Table 3 shows that there is approximately a 2-fold increase in BAL-derived NPs over ambient laboratory contamination.

**Figure 5:**
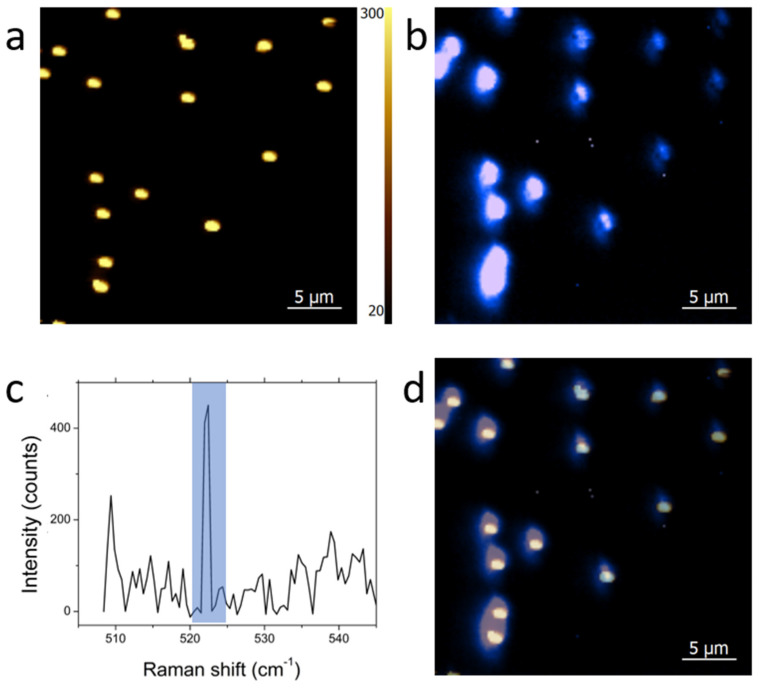
TERS of spiked recovery experiments on 500 nm-diameter Si spheres: (**a**) AFM height imaging, (**b**) TERS integrated intensity of the 521 cm^−1^ Si peak in (**c**), and (**d**) the overlap of AFM and TERS images shows 16 Si particles in the 30 × 30 µm^2^ area.

To verify that TERS can accurately quantify the NPs in BAL, we performed spiked recovery experiments on 500 nm-diameter Si spheres (Figure 5). We counted a total of 16 particles in both the 30 × 30 µm^2^ area using AFM (Figure 5a) and using TERS imaging (Figure 5b) of the Si Raman peak at 521 cm^−1^ (Figure 5c). The overlap of the AFM and TERS images in Figure 5d shows the perfect correlation of all 16 particles, which demonstrates 100% efficiency of detection using TERS.

## 4. Discussion

Our results demonstrate the successful measurements of strong TERS signals from more than 60 different locations in a 50 µm × 50 µm area without the mixing of components or fluorescence background interference. More than half of these locations correspond to the edge area of the sample coffee-ring region. The area of the coffee ring was estimated as 61,575 µm^2^ based on the total dried droplet circular spot size of ~1 mm in diameter and the 20 µm ring width. The near-field sampling area formed by the tip-sample contact can be assumed as a circle area with a 20 nm radius. The detected particles were few in number (60 particles were detected in 2500 measurement locations on a spatial grid of 50 × 50 points), resulting a ~2.5% occupation of the whole sampling area. The resulting undersampling due to the 1 µm step size can be resolved by increasing the scanning range of the imaging areas and detecting a larger number of NPs. This clinical study is, however, outside the scope of our work and will be performed in the future.

Since we did not use the nanoscale step size in our TERS imaging scans, we have an upper bound on the particle size of 1 µm, which is our scanning step size. Therefore, we define our NPs as sub-micron particles. Assuming that most of the NPs were concentrated in this coffee-ring area and using the circular area of the near-field laser focus of a 20 nm radius, we estimated the total nanoparticle density in BAL to be ~50 billion particles per liter. Figure 4c shows that most of these particles were plastic NPs. This is in agreement with previously reported concentrations of billions of NPs found in food and the environment [19,20]. Likewise, we estimated the total microplastic particulate density within the BAL sample to be ~200 MPs/L, similar to previously reported concentrations of 92 particles/L [9] and 320 particles/L [39].

Of the various NPs identified through TERS characterization, PS was the only plastic observed within the sample area and not in the controls. PS is widely used in multitudinous industrial applications, ranging from commercial shipping, biomedical applications, construction, and textile applications and is commonly found in the commercial packaging foam [40]. The presence of PS NPs in BAL samples may be attributed to the inhalation of aerosolized PS particulates, which have been observed in various environments, including the use of face masks [41,42]. Aerosolized PS NPs have recently shown detrimental effects on pulmonary tissues [43].

Similarly, PU is ubiquitously employed in various applications, including biomedical technology, textile elastomers, protective coatings, and sealants [44,45]. We found PU both in sample and control areas. The presence of PU NPs in BAL samples may be attributed to its ubiquitous applications in daily life and its use in various biomedical devices, the mechanical shearing of commercial products that produces nano-particulates, and aerosolized PU pollution [45,46]. Similarly to the PS NPs, the presence of PU NPs in BAL samples may potentially damage pulmonary tissues [46].

Various NPs such as PE, PP, PMMA, SIS, PA, PVC, and ABS were identified both in the control area and on the edge of the BAL droplet. The edge area also had PES, PEVA, and butadiene-styrene, which were not identified in the control area. Both PES and PEVA have biomedical applications, as PES is often employed as a membrane material for hemodialysis and medical device manufacturing, while PEVA is utilized in transdermal drug-delivery systems, intravenous fluid storage, and antimicrobial catheter production [47,48,49]. In contrast, butadiene-styrene (SB) is a synthetic rubber, often employed in the production of automotive tires, footwear, electrical cable insulation, sealants, and textiles [50]. The multitudinous use of both PES and PEVA in various biomedical applications indicates that their presence may result from contamination throughout sample preparation or clinical environmental contamination vectors. Complex chemical processes may convert ABS into butadiene-styrene or both may show similar TERS spectra through spectral overlap.

TERS identified various synthetic pigments within control, edge, and sample areas. More pigments were found in the control compared to the edge/sample areas. Mostly blue pigments were found in the edge and sample areas, while the control area also contained other pigments. The specific pigment types were identified using the IDFinder software with the spectral library from Horiba Scientific, as described in the Methods Section. Several close ID matches were found, and the matches with the best spectral overlap were chosen. However, in some cases, there were several close matches identified. Therefore, the actual pigment types could vary from the ones specified in Table 2 and Table 3. However, the close matched pigments typically had similar chemical compositions and similar corresponding sources of industrial applications. For example, copper phthalocyanine (CuPc), Cromophtal blue, Astra blue, and Hostaperm green are phthalocyanine derivatives, which are utilized as both plastic pigments and dyes in various textiles [51,52]. Hostaperm green, in particular, is often employed as a pigment for paint or coatings, indicating a potential contamination vector through aerosolized paint particulates or dust [53]. Similarly, Drimaren is a derivative azo dye that is often employed in textile dying, often with textiles produced from cellulose polymer scaffolds, such as cotton or rayon [54]. Therefore, the presence of Drimaren pigments within the control area is likely due to the contamination resulting from textile sources during sample collection or preparation. Similarly, the most likely origin of other blue pigments within each corresponding substrate area is due to the pigments’ varying applications in plastic and textile coloring. CuPc, Methylene blue, Victoria blue, and Cromophtal blue have been employed in both plastic and textile coloring applications [52,55,56]. Therefore, the presence of such pigments within various substrate areas is likely a result of contamination from the observed microplastic species or textile sources. In contrast, although Astra blue is a copper phthalocyanine derivative, it is not typically employed in textile coloring, but rather in various cellular-staining protocols [57]. Astra blue is a potential misidentification of the pigment from the other copper-phthalocyanine-derivative pigments due to the spectral overlap as discussed above.

TERS also identified various organic polymers, including cholesteryl benzoate, cotton, and cellulose, as well as minerals such as carbon and hematite (Table 2 and Table 3). Cholesteryl benzoate has applications in liquid crystal displays, thermochromic materials, and other material sciences [58]. The origin of cholesteryl benzoate in the control area is unclear and is rather likely to originate from the surrounding electronic instrumentation in the laboratory environment. Although esterified cholesterol derivates have been isolated from in vivo tissue studies, cholesteryl benzoate is a synthetic ester that is not endogenously produced within the body, making an internal biological process highly unlikely as the origin of the observed cholesteryl benzoate signal.

Comparatively, both the cotton and cellulose spectral signals from the edge sample area are readily explained by the likelihood of sample exposure from textiles or textile-related particulates. Cotton, which is mostly comprised of cellulose fibers, is ubiquitously used in textile manufacturing [59,60]. Thus, it is probable that textile particulates composed of cotton or cellulose fiber materials originate from BAL samples or laboratory-personnel-worn textiles. Previous studies of MP analyses of BAL samples reported the presence of textile fibers [9]. We also found several colored fibers during the MP analysis of our BAL samples but were unable to identify their composition using Raman spectroscopy due to the large fluorescence background, which is typical for colored samples. As mentioned above, the advantage of TERS is that it can suppress fluorescence, and, therefore, TERS may detect colored cotton or cellulose nanoparticles that originated from the larger textile fibers.

Hematite has predominant applications in iron extraction, steel production, and electronics, although contemporary efforts utilize hematite in various nanotechnologies [61,62]. Thus, the observed hematite nanoparticles may originate from nanotechnologies or electronic technologies within the laboratory environment or the mechanical sheering of laboratory steel infrastructure, thereby resulting in the formation of iron particulates, which then undergo oxidation forming hematite particulate contaminants. Recent research on microplastics in human urine and kidney tissue also found the presence of copper phthalocyanine and hematite microparticles [63].

TERS can be further extended to the study of other body fluids and can be combined with other techniques, such as microfluidic aptamer-based sensors, for the multi-modal detection of other components, such as toxic heavy metal ions [64]. The nanoscale imaging of aptamers using TERS was previously demonstrated [65]. Our results correspond with data from previous studies, reinforcing the application of TERS in BAL analysis. Primarily, MPs, NPs, and various synthetic fibers were observed, which have likewise been reported in previous works [9,10,11,34,39]. Such findings suggest the success of TERS as a valid sensing modality for detecting various analytes of distinct chemical classifications, in mixed-sample sensing. Furthermore, the success of the TERS identification of target analytes within the provided BAL sample demonstrates that TERS circumvents the current challenges of pre-existing techniques, particularly concerning interference from biological aqueous environment sand fluorescence interference. Thus, as confirmed in this report, TERS provides an improved modality for the sensing of diverse analyte species, with greater feasibility compared to preexisting methodologies. These features underscore the potential applicability of TERS for the clinical detection of various analytes across diverse biofluids, as validated by the current study’s findings.

## 5. Conclusions

In summary, we showed the first application of TERS to the study of NPs in BAL human samples. The comparison of the conventional Raman spectroscopy and TERS provided evidence of the potential origin of the nanoscale NPs from the corresponding microscale MPs. A larger number of nanoparticle compositions were identified compared to microparticle compositions due to the suppression of the fluorescence background in TERS and plasmonic signal enhancement. A comparison of the TERS of BAL samples with the environmental control showed large contamination of the substrate by NPs, pigments, biopolymers, and minerals, which requires careful analysis when attempting to understand the effects of NPs on health.

## Figures and Tables

**Table 1:** Relative strengths and weaknesses of AFM/TERS versus other methods of MP/NP analysis. * No chemical specificity; only topography. ** Water interference in FTIR; fluorescence in Raman.

Feature	AFM/TERS	SEM	FTIR	Raman
High Spatial Resolution	✓ 1 nm	✓ 1 nm	10 μm	1 μm
High Chemical Specificity	✓		✓	✓
No Sample Prep	✓		✓	✓
Detects Microplastics	✓	✓ *	✓	✓
Detects Nanoplastics	✓	✓ *		
No Background **	✓	✓		

**Table 2:** Nanoparticle compositions identified in the BAL sample, droplet edge, and SiO_2_/Si substrate control areas.

Material Type	Control	Edge	Sample
Mineral		Hematite	
Org polymer	Cholesteryl benzoate	Cotton, cellulose	Cotton
Pigment	CuPc, Drimaren, Hostaperm green, Irisol blue, Methylene blue, Victoria blue	Cromophtal blue	Astra blue
Plastic	SIS, PVC, PU, PP, PMMA, PEVA, PES, PE/PP, PA, ABS	SIS, PVC, PU, PS, PP, PES, PE, PA, SBR, ABS	PU, PS

**Table 3:** List of the nanoparticle composition identified in the clean SiO_2_/Si substrate control area.

Material Type	Chemicals
Plastic	PU, PA, PVC, SIS, PE/PP, PEVA
Mineral	Carbon
Org Polymer	Cellulose
Pigment	Indigo, Pigment orange 48, Irisol blue, Cromophtal blue, Astra blue, Pigment orange 36 Gamboge, Zapon blue, Pigment violet 19

## Data Availability

The raw data supporting the conclusions of this article will be made available by the authors on request.
